# Language disparity is not a significant barrier for time-sensitive care of acute ischemic stroke

**DOI:** 10.1186/s12883-020-01940-9

**Published:** 2020-10-02

**Authors:** Noriko Anderson, Afra Janarious, Shimeng Liu, Lisa A. Flanagan, Dana Stradling, Wengui Yu

**Affiliations:** 1 Department of Neurology, University of California, San Francisco, CA USA; 2 Department of Neurology, University of California, Irvine, CA USA; 3 Orange, CA USA

**Keywords:** Acute ischemic stroke, Language disparity, Benchmark, Outcome, Thrombolysis

## Abstract

**Background:**

Language barriers were reported to affect timely access to health care and outcome. The aim of this study was to investigate the effect of language disparity on quality benchmarks of acute ischemic stroke therapy.

**Methods:**

Consecutive patients with acute ischemic stroke at the University of California Irvine Medical Center from 2013 to 2016 were studied. Patients were categorized into 3 groups according to their preferred language: English, Spanish, and other languages. Quality benchmarks and outcomes of the 3 language groups were analyzed.

**Results:**

Of the 928 admissions, 69.7% patients recorded English as preferred language, as compared to 17.3% Spanish and 13.0% other languages. There was no significant difference in the rate of receiving intravenous thrombolysis (24.3, 22.1 and 21.0%), last-known-well to door time, door-to-imaging time, door-to-needle time, and hospital length of stay among the 3 language groups. In univariate analysis, the other languages group had lower chance of favorable outcomes than the English-speaking group (26.3% vs 40.4, *p* < 0.05) while the Spanish-speaking group had lower mortality rate than English-speaking group (3.1% vs 7.7%, *p* = 0.05). After adjusting for age and initial NIHSS scores, multivariate regression models showed no significant difference in favorable outcomes and mortality between different language groups.

**Conclusion:**

We demonstrate no significant difference in quality benchmarks and outcome of acute ischemic stroke among 3 different language groups. Our results suggest that limited English proficiency is not a significant barrier for time-sensitive stroke care at Comprehensive Stroke Center.

## Background

Intravenous thrombolysis (IVT) with recombinant tissue plasminogen activator (tPA) remains the only proven medical therapy for acute ischemic stroke (AIS) [1]. An analysis of pooled data from clinical trials found that earlier the patient receives tPA, better the chances of recovery [2]. IVT rates in *Get-With-The-Guideline (GWTG)-*Stroke-participating hospitals have improved significantly over time [3]. However, a recent study reported that up to 18% potentially eligible patients were not being treated with IVT [4]. After adjusting for stroke severity, minorities remained more likely to not receive treatment [4, 5]. Thus, identification of the practice hurdles and development of improved system of care should be a high priority for stroke research.

One of the possibilities for lower IVT rates in minorities could be language barriers. In the United States, 18.7% of the residents speak a language other than English at home and 8.4% residents have limited English proficiency [6]. In California, approximately 30% patients have limited English proficiency [7]. Language barriers were reported to affect timely access to health care and patient-physician communications [8–12].

Rostanski et al. investigated the language discordance between patients and physicians and found no effect of language discordance on door-to-imaging (DTI) time and door-to-needle (DTN) time [13]. Luan et al. analyzed single center data of 3894 AIS patients and found no significant difference in IVT rate between English and non-English-preferring patients [14]. However, previous studies reported conflicting results on the outcomes of patients with language disparity. Shah et al. analyzed data from the Registry of the Canadian Stroke Network and showed reduced mortality and better performance on some quality of care measures in patients with language barriers [15]. In contrast, Kilkenny et al. examined data from the Australian Stroke Clinical Registry and showed that patient requiring interpreters had comparable discharge outcomes but poorer Health Related Quality of Life than patients not needing interpreters [16].

In this study, we sought to appraise whether language disparity affects IVT rate, time-sensitive quality benchmarks, and outcomes of AIS at a Joint Commission-certified Comprehensive Stroke Center in South California, where approximately 30% patients have limited English proficiency [7].

## Methods

Patient population and study protocol: Consecutive patients admitted for AIS at the University of California Irvine Comprehensive Stroke Center from January 1st, 2013 to December 31st, 2016 were included in the study. The patient list was generated from the prospectively maintained American Heart Association (AHA) “*Get-With-The-Guideline (GWTG)® Stroke*” program at our hospital. The registry uses a web-based patient management tool to collect clinical data on consecutively admitted patients, to provide decision support, and to enable real-time online reporting features [17]. Patients with TIA, stroke mimics, subacute stroke, and inpatient stroke were excluded. The following data were collected from the registry and electronic medical record by experienced neurologists (AJ, NA, and SL): patient demographics, NIHSS scores, initial blood pressure (BP), body mass index (BMI), last-known-well (LKW)-to-door time (LKW-DT), DTI time, DTN time for IV tPA, hospital length of stay (LOS), and modified Rankin Scale (mRS) scores at hospital discharge. Laboratory results, including comprehensive metabolic panel, serum levels of lipids and glycol-hemoglobin A1c (HbA1c) were also collected. A mRS score ≤ 2 was defined as favorable outcome. Patients were categorized into 3 groups according to their preferred language: English, Spanish, and other-languages (including Vietnamese, Korean, Mandarin, Cantonese, Japanese, Punjabi, Romanian, Arabic, and Tagalog).

We have standard protocols for the management of patients with limited English proficiency. When the provider encounters language barrier, interpreter service will be requested for assistance. Spanish and Vietnamese interpreters were available Monday through Friday, 7:30 a.m. to 4:30 p.m. in the emergency room (ER). Whenever on-site interpreter was not available, tele-interpreter service was used for patient/provider communication. Telephonic or in-person interpreter was available 24/7 via phones or Video Remote Interpreter carts. Other healthcare providers and family members were used only when official interpreter service was declined or unavailable.

### Statistical analysis

Categorical variables were expressed as frequencies and percentages (%), and continuous variables as mean plus standard deviation (SD). Tukey’s Studentized Range Test was used to estimate the differences in means with 95% confidence interval (CI) between different language groups. Univariate logistic regression analysis was performed to test the crude odds ratios (OR) (95% CI) of the benchmarks comparing with English-speaking patients. Multivariate logistic regression models were performed to determine the adjusted OR (95% CI) of favorable outcome (mRS 0–2) and mortality at hospital discharge, in association with the preferred language. In the multivariate logistic regression models, we adjusted for potential confounding factors (i.e., age and initial NIHSS scores). The SAS statistical software (version 9.4; SAS Institute, Cary, NC, USA) was used to perform the data analysis.

## Results

Nine-hundred twenty-eight AIS admissions were identified during the study period. In this single center cohort, 69.7% patients recorded English, 17.3% Spanish, and 13.0% other languages as preferred language. The ethnic compositions of the English group were 79.6% White, 12.7% Hispanic, 12.7% Asian, and 3.2% Black. The baseline characteristics of the 3 groups were summarized in Table 1. Compared with English-speaking group, Spanish-speaking patients were significantly younger (65 ± 14 vs. 69 ± 16, *p* < 0.05) while other language-speaking patients were significantly older (75 ± 12 vs. 69 ± 16, *p* < 0.05) and had more women (55.5% vs. 45.1%, *p* < 0.05). Both Spanish-speaking and other language-speaking groups had significantly lower BMI than the English-speaking group. In contrast, the other languages-speaking group had significantly higher levels of HbA1c than English-speaking and Spanish-speaking groups. There was no significant difference in initial blood pressures and LDL cholesterol levels at admission among the 3 groups.

**Table 1:** Baseline characteristics of the AIS patients

	English	Spanish	Other language	Spanish vs. English	Other language vs. English
	*n* = 646	*n* = 163	*n* = 119	Difference (95% CI)	Difference (95% CI)
Age, y, mean (SD)	69 (16)	65 (14)	75 (12)	−4.5 (−7.7 - -1.4) *	6.2 (2.6–9.8) *
Female, n (%)	291 (45.1%)	79 (48.5%)	66 (55.5%)	1.1 (0.8–1.6)	1.5 (1.0–2.2) *
BMI, mean (SD)	27.0 (6.0)	28.5 (5.9)	24.5 (4.5)	1.5 (0.3–2.7) *	−2.5 (− 3.9 - -1.1) *
SBP, mmHg, mean (SD)	161 (33)	164 (33)	166 (32)	2.8 (−4.0 - 9.7)	4.9 (− 2.9 - 12.6)
DBP, mmHg, mean (SD)	88 (19)	87 (17)	87 (18)	7.5 (−0.7 - 15.7)	4.2 (−5.4 - 13.9)
LDL-c, mmol/L, mean (SD)	2.6 (1.0)	2.8 (1.0)	2.7 (0.9)	0.2 (0.0–0.4)	0.1 (−0.1 - 0.4)
HbA1c, %, mean (SD)	6.6 (3.9)	7.5 (2.3)	12.6 (59.4)	0.9 (−3.6 - 5.3)	6.0 (0.7–11.3) *

*Abbreviations: CI* confidence interval, *SD* standard deviation, *BMI* body mass index, *SBP* systolic blood pressure, *DBP* diastolic blood pressure, *LDL-c* low-density lipoprotein cholesterol, *HbA1c* glycated hemoglobin A1c

Differences in means between groups were calculated by the Tukey’s Studentized Range Test. Odds ratios in dichotomous variables compared Spanish or other languages with English and were tested in the unadjusted logistic regression models

*P < 0.05

The stroke severity and quality benchmarks of stroke care were shown in Table 2. There was no significant difference in initial NIHSS scores, DTI time, IVT rate, and LOS among the 3 language groups. Compared to English-speaking group, the other language-speaking group had lower chances of favorable outcomes (26.3% vs 40.0%; OR, 0.5 [95% CI, 0.4–0.8]; *p* = 0.01) while the Spanish-speaking group had lower mortality rate (3.1% vs 7.7%, OR, 0.4 [95 CI, 0.1–1.0]; *p* = 0.04).

**Table 2:** Stroke severity and quality benchmarks of stroke care among the three language groups

	English	Spanish	Other languages	Spanish vs. English	Other languages vs. English
	*n* = 646	*n* = 163	*n* = 119	Difference or OR (95% CI)	Difference or OR (95% CI)
Initial NIHSS scores, mean (SD)	9 (8)	8 (8)	10 (9)	−1.0 (− 2.7 - 0.7)	1.3 (− 0.6 - 3.3)
DTI time (mins)	58 (100)	65 (91)	56 (93)	7.4 (− 12.6 - 27.5)	−2.0 (− 24.8 - 20.8)
IV tPA received, n (%)	157 (24.3%)	36 (22.1%)	25 (21.0%)	0.9 (0.6–1.3)	0.8 (0.5–1.3)
LOS, days, mean (SD)	5 (6)	6 (7)	6 (6)	0.8 (− 0.4 - 2.0)	0.5 (− 0.9 - 1.9)
mRS (0–2) at discharge, n (%)	247 (40.0%)	61 (38.6%)	31 (26.3%)	1.0 (0.7–1.4)	0.5 (0.4–0.8) ^*^
Death at hospital, n (%)	50 (7.7%)	5 (3.1%)	7 (5.9%)	0.4 (0.1–1.0) ^*^	0.7 (0.3–1.7)

*Abbreviations: CI* confidence interval, *DTI* door-to-imaging, *IQR* interquartile range, *LOS* length of stay, *mRS* modified Rankin Scale, *NIHSS* National Institutes of Health Stroke Scale, *SD* standard deviation, *tPA* tissue plasminogen activator

Differences in means between groups were calculated by the Tukey’s Studentized Range Test

Odds ratios in dichotomous variables were tested in the unadjusted logistic regression models

*P < 0.05

To eliminate any potential confounding factors, multivariate logistic regression model was performed to assess the relationship between preferred languages and clinical outcomes (Table 3). After adjusting for age and initial NIHSS scores, there was no significant difference in favorable outcome at hospital discharge between the 3 language groups. With each point increase in NIHSS score or each year increase in age, the patients were 14% or 3% less likely to have favorable outcome at hospital discharge, respectively. Likewise, after adjusting for age and initial NIHSS scores, the Spanish-speaking group did not have lower mortality rate than the English-speaking group (OR, 0.44 [95% CI, 0.17–1.18]; *p* = 0.10). Higher initial NIHSS score was correlated with higher mortality rate (OR, 1.15[95% CI, 1.11–1.19]; *p* < 0.0001). To follow the Principle of Parsimony in variable selections, we stopped adding other variables in the multivariable logistic regression analysis afterwards.

**Table 3:** Multivariate logistic regression analysis of the relationships between preferred language and clinical outcome

	Odds ratio	95% CI	*P value*
**Favorable outcome (mRS 0–2)**			
Language			
Spanish vs. English	0.77	0.52–1.15	0.21
Other language vs. English	0.68	0.42–1.11	0.12
Initial NIHSS scores	0.86	0.84–0.89	< 0.0001
Age	0.97	0.96–0.98	< 0.0001
**Death at hospital**			
Language			
Spanish vs. English	0.44	0.17–1.18	0.10
Other language vs. English	0.55	0.23–1. 33	0.18
Initial NIHSS scores	1.15	1.11–1.19	< 0.0001
Age	1.01	0.99–1.03	0.36

*Abbreviations: CI* confidence interval, *mRS* modified Rankin Scale, *NIHSS* National Institutes of Health Stroke Scale

Among the 218 patients who were treated with IVT, there was no significant difference in LKW-DT, DTI time, DTN time, hospital LOS, favorite outcome and mortality between English and Spanish or other language groups (Table 4). Language disparity has no significant effect on time-sensitive quality benchmarks of IVT.

**Table 4:** Quality benchmarks and outcome of patients who received IV tPA

	English	Spanish	Other language	Spanish vs. English	Other language vs. English
	*n* = 157	*n* = 36	*n* = 25	Difference or OR (95% CI)	Difference or OR (95% CI)
LKW-TD time, mean (SD)	77 (56)	101 (134)	85 (56)	23.8 (−8.8 - 56.3)	8.2 (−29.7 - 46.1)
DTI time, mean (SD)	20 (19)	18 (13)	19 (16)	−1.8 (−9.4 - 5.8)	− 1.5 (− 10.4 - 7.4)
DTN time, mean (SD)	59 (32)	48 (17)	58 (33)	2.5 (−2 - 4)	−10.8 (− 27.4 - 5.7)
Hospital LOS, mean (SD)	6 (6)	7 (8)	6 (4)	1.0 (−1.8 - 3.8)	0.1 (− 3.1 - 3.4)
mRS (0–2) at discharge, n (%)	63 (40.9%)	13 (36.1%)	11 (44.0%)	0.8 (0.4–1.7)	1.1 (0.5–2.7)
Death at hospital, n (%)	12 (7.6%)	1 (2.8%)	2 (8.0%)	0.4 (0.2–1.0)	0.8 (0.3–1.7)

Differences in means between groups were calculated by the Tukey’s Studentized Range Test. Odds ratios (OR) in dichotomous variables were tested in the unadjusted logistic regression models

*Abbreviations: CI* confidence interval, *DTI* door-to-imaging, *DTN* door-to-needle, *LKW-TD* Last-known-well to door, *mRS* modified Rankin Scale

*OR* odds ratios, *SD* standard deviation

## Discussion

Ethnic minorities in the United States may have limited English proficiency and substandard care due to language barriers. However, previous studies have showed conflicting results on the impact of language barriers. Emergency medical service usage was actually reported higher in the Spanish-speaking patients than the English-speaking patients treated with IVT [18]. A retrospective single-center study showed that language discordance was not associated with acute stroke misdiagnosis among patients treated with IVT [19]. Another study reported reduced mortality in patients with language barriers [13]. The possible explanations for reduced mortality, however, were likely due to inclusion of patients with TIA and family desire for aggressive intervention over quality of life [13, 20].

The AHA quality improvement initiatives and the Comprehensive Stroke Center certification have improved stroke care in recent years (https://www.jointcommission.org/certification/advanced_certification_comprehensive_stroke_centers.aspx) [3, 21–23], Patients with limited English proficiency may receive similar quality of care as English-speaking patients at Comprehensive Stroke Centers partly due to the rigorous re-certification requirement for ongoing performance improvement in stroke care. The Comprehensive Stroke Center re-certification is conducted by the Joint Commission every 2 years. At the re-certification site visit, the surveyors assess the ongoing quality improvement in 18 core quality measures (including DTN time) and opportunities for further improvement (e.g., language disparity and availability of interpreter service). The re-certification citations on any deficiency or opportunities for improvement often lead to additional support for better stroke care, such as 24/7 availability of interpreter service and hiring of additional fellows or nurse practitioner.

In the current study, we demonstrate that there is no significant difference in quality benchmarks, IVT rate, and outcomes of AIS among 3 language groups at an academic Comprehensive Stroke Center.

The diversity of healthcare providers and interpreter/tele-interpreter service may have overcome language barriers. Our IVT rate and DTN time are comparable or better than what were reported recently from other Comprehensive Stroke Centers [14, 19, 21, 22, 24]. We have also shown that increased age and higher initial NIHSS scores were associated with unfavorable outcomes at hospital discharge.

The strengths of the current study include: 1). The study was performed at a Comprehensive Stroke Center in a region with significant language disparity. 2). Our data reflects current standard care at academic Comprehensive Stroke Centers in the United States. 3). Univariate and multivariate regression models were performed to investigate the language disparity as potential barrier for time-sensitive quality benchmarks and outcomes.

Our study has a few limitations. First, due to lack of long-term follow-up data, we were only able to investigate the effects of language disparity on outcomes at hospital discharge. Second, the retrospective study was unable to determine the specific contribution of interpreters and tele-interpreter service to the improvement of stroke care in patients with language barriers. Third, at our medical center, all ER personnel and providers speak English as official language. Some of them may speak Spanish or other languages fluently. However, in this retrospective study, we do not have data on languages spoken by hospital staff and how fluently family members are who provided the history and helped coordinate care. Finally, patients with a preferred language other than English do not necessarily communicate ineffectively in English. In this retrospective study, we do not have information on how patients’ proficiency in English was assessed and cannot determine how many patients indeed needed language interpretation.

## Conclusion

In this single center study, we demonstrate no significant difference in IVT rate, quality benchmarks, and outcome of AIS among 3 language groups. Our findings suggest that limited English proficiency is not a significant barrier for acute stroke care at Comprehensive Stroke Center.

## Data Availability

The datasets generated and analysed during the current study are not publicly available, but data may be available from the corresponding author on reasonable request.

## Abbreviations

AIS: acute ischemic stroke
BMI: body mass index;
CI: confidence interval
DBP: diastolic blood pressure
DTI: door-to-imaging
DTN: door-to-needle
HbA1c: glycated hemoglobin A1c
IQR: interquartile range
LDL-c: low-density lipoprotein cholesterol
LKW-TD: Last-known-well-to-door
LOS: length of stay
mRS: modified Rankin scale
NIHSS: National Institutes of Health stroke scale
OR: odds ratios
SBP: systolic blood pressure
SD: standard deviation
tPA: tissue plasminogen activator
